# The Protective Role of miR-130b-3p Against Palmitate-Induced Lipotoxicity in Cardiomyocytes Through PPARγ Pathway

**DOI:** 10.3390/ijms252212161

**Published:** 2024-11-13

**Authors:** Elena Alonso-Villa, Alipio Mangas, Fernando Bonet, Óscar Campuzano, Maribel Quezada-Feijoo, Mónica Ramos, Carlos García-Padilla, Diego Franco, Rocio Toro

**Affiliations:** 1 Biomedical Research and Innovation Institute of Cadiz (INiBICA), Research Unit, Puerta del Mar University Hospital, 11009 Cádiz, Spain; elena.alonso@gm.uca.es (E.A.-V.); alipio.mangas@uca.es (A.M.); fbonetmartinez@gmail.com (F.B.); 2 Medicine Department, School of Medicine, University of Cadiz, 11002 Cádiz, Spain; 3 Lipid and Atherosclerotic Unit, Internal Medicine Department, Puerta del Mar University Hospital Cardiology Service, 11009 Cádiz, Spain; 4 Hospital Josep Trueta, University of Girona, 17007 Girona, Spain; ocampuzano@idibgi.org; 5 Cardiovascular Genetics Center, University of Girona-IDIBGI, 17190 Girona, Spain; 6 Centro de Investigación Biomédica en Red, Fisiopatología Obesidad y Nutricion (CIBEROBN), Instituto de Salud Carlos III, 28029 Madrid, Spain; 7 Cardiology Department, Hospital Central de la Cruz Roja, 28003 Madrid, Spain; maribelquezadafeijoo2000@gmail.com (M.Q.-F.); monica.ramos81@gmail.com (M.R.); 8 Medicine School, Alfonso X el Sabio University (UAX), 28691 Madrid, Spain; 9 Cardiovascular Research Group, Department of Experimental Biology, University of Jaen, 23071 Jaen, Spain; cgpadill@ujaen.es (C.G.-P.); dfranco@ujaen.es (D.F.); 10 Medina Foundation, Technology Park of Health Sciences, 18016 Granada, Spain

**Keywords:** miR-130b-3p, PPARγ, lipotoxicity, dilated cardiomyopathy, mitochondrial oxidative stress, endoplasmic reticulum stress

## Abstract

Excess lipid accumulation in the heart is associated with lipotoxicity and cardiac dysfunction due to excessive fatty acid oxidation. Peroxisome proliferator-activated receptor gamma (PPARγ) modulates the expression of key molecules involved in the FA metabolic pathway. Cardiomyocyte-specific overexpression of PPARγ causes dilated cardiomyopathy associated with lipotoxicity in mice. miR-130b-3p has been shown to be downregulated in the plasma of idiopathic dilated cardiomyopathy patients, but its role in modulating cardiomyocyte lipotoxicity via PPARγ remains unclear. Our objective was to investigate the protective role of miR-130b-3p against palmitate-induced lipotoxicity in cardiomyocytes through the modulation of the PPARγ signaling pathway. Human cardiomyoblasts were treated with palmitate. Intracellular lipid accumulation and expression of PPARγ and its downstream targets (*CD36*, *FABP3*, *CAV1*, *VLDLR*) were analyzed. Mitochondrial oxidative stress was assessed via MitoTracker Green and Redox Sensor Red staining and expression of *CPT1B* and *SOD2*. Endoplasmic reticulum stress and apoptosis were determined by examining GRP78, *ATF6*, XBP1s, *CHOP*, and caspase-3 expression. miR-130b-3p overexpression was achieved using transfection methods, and its effect on these parameters was evaluated. Luciferase assays were used to confirm PPARγ as a direct target of miR-130b-3p. Palmitate treatment led to increased lipid accumulation and upregulation of PPARγ and its downstream targets in human cardiomyoblasts. Palmitate also increased mitochondrial oxidative stress, endoplasmic reticulum stress and apoptosis. miR-130b-3p overexpression reduced PPARγ expression and its downstream signaling, alleviated mitochondrial oxidative stress and decreased endoplasmic reticulum stress and apoptosis in palmitate-stimulated cardiomyoblasts. Luciferase assays confirmed PPARγ as a direct target of miR-130b-3p. Our findings suggest that miR-130b-3p plays a protective role against palmitate-induced lipotoxicity in cardiomyocytes by modulating the PPARγ signaling pathway.

## 1. Introduction

The adult heart is a highly dynamic organ that derives 50–70% of its ATP requirements from the oxidation of fatty acids (FAs) [1]. Approximately 80% of free FAs uptake in cardiomyocytes occurs via a protein-mediated transport mechanism [1]. Several proteins are involved in this process: fatty acid translocase (FAT/CD36), acid transport proteins (FATP) 1 and 6 or cytosolic fatty acid binding protein (cFABP). FAT/CD36 downstream signaling also involves caveolin 1 (CAV1), which is a specific fatty acid transporter and very-low-density-lipoprotein receptor (VLDLR) [2]. Then, FAs are converted in acyl-CoA by acetyl-CoA synthetase and transferred to the mitochondrial matrix by the carnitine shuttle constituted by carnitine-palmitoyltransferase 1 (CPT1b) for FA oxidation (FAO) [3].

Several studies indicate that PPAR transcription factors, highly expressed in the heart, regulate FA transporters [4,5]. PPARs control energy metabolism, inflammatory responses, and lipid homeostasis by binding to ligands and cofactors PPARs. This protein family comprises three isoforms, PPARα, PPARγ, and PPARδ, that heterodimer with the retinoid X receptor to bind to peroxisome proliferator response elements and activate gene transcription [6]. PPARα and PPARδ regulate mitochondrial FAO, glycolysis and FA synthesis in cardiomyocytes. On the other hand, PPARγ promotes FA absorption, triglyceride formation and storage in lipid droplets [7].

Cardiomyocyte lipid accumulation due to excess FA uptake leads to lipotoxicity associated with dilated cardiomyopathy (DCM; OMIM #115200). DCM is a cardiac condition characterized by enlargement of the left ventricle or both ventricles, accompanied by impaired systolic function. Genetic factors play a significant role in the etiology of DCM, with current estimates suggesting that 35–50% of cases have a genetic basis [8]. The cardiac-specific deficiency of very long-chain acyl-CoA dehydrogenase, a catalytic enzyme for mitochondrial oxidation, leads to cardiomyopathy [9]. Patients with DCM and ischemic cardiomyopathy showed increased lipid accumulation in certain left ventricle areas [10]. Concomitantly, integrated bioinformatics analysis of gene expression in DCM revealed potential diagnostic and preventive markers for DCM such as *PLIN2*, a marker of FA storage [11].

MicroRNAs (miRNAs) belong to the small, single-stranded and non-coding RNAs group, with an average length of 18–22 nucleotides. They exert a negative regulatory influence on gene expression at the post-transcriptional level [12]. Multiple miRNAs have been identified to play key roles in DCM by regulating cellular mechanisms such as ROS production, endoplasmic reticulum (ER) stress, mitochondrial dysfunction, cardiac fibrosis, hypertrophy or cardiac inflammation [13]. We previously observed downregulation of miR-130b-3p in the plasma of idiopathic DCM patients with reduced ejection fraction compared to healthy and ischemic DCM patients [13]. miR-130b-3p has been shown to inhibit FA accumulation in pigs and bovine species by decreasing *PPARγ* expression [14,15,16]. Consistent with this, Lee et al. reported that miR-130b-3p negatively regulates *PPARγ* expression by targeting the 3’UTR region in carcinoma cells and inhibited adipogenesis by miR-130b overexpression [17]. Furthermore, recent studies suggest that miR-130 inhibition alleviates infarct-induced myocardial injury by promoting *PPARγ* expression [18]. However, whether miR-130b-3p modulates *PPARγ* in the heart remains unstudied.

In the present study, we aim to unravel the role of miR-130b-3p on *PPARγ* regulation and FA metabolism in human cardiomyocytes.

## 2. Results

### 2.1. Overexpression of miR-130b-3p Alleviated Hight Palmitate-Induced Lipotoxicity In Vitro

**Figure 1:**
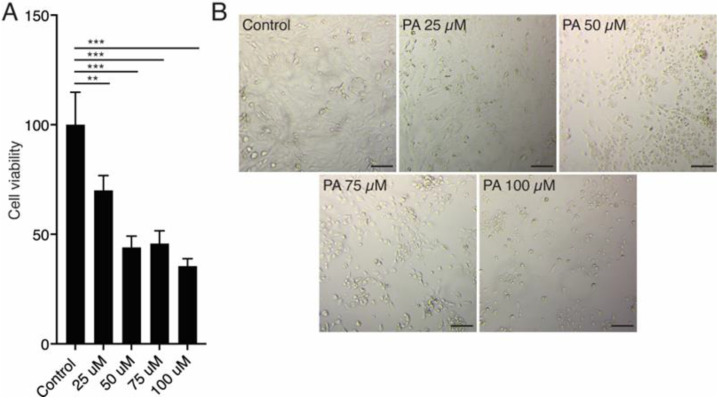
MTS cell viability assay results of different PA treatment. (**A**) AC16 treated with different PA concentrations (0, 25, 50, 75, 100 μM) for 24 h. (**B**) Cell morphological changes were assessed after PA treatment for 24 h. ** *p* < 0.01, *** *p* < 0.005. Error bars represent SDs. Scale bars = 100 μm. PA. Abbreviations: PA, palmitate.

**Figure 2:**
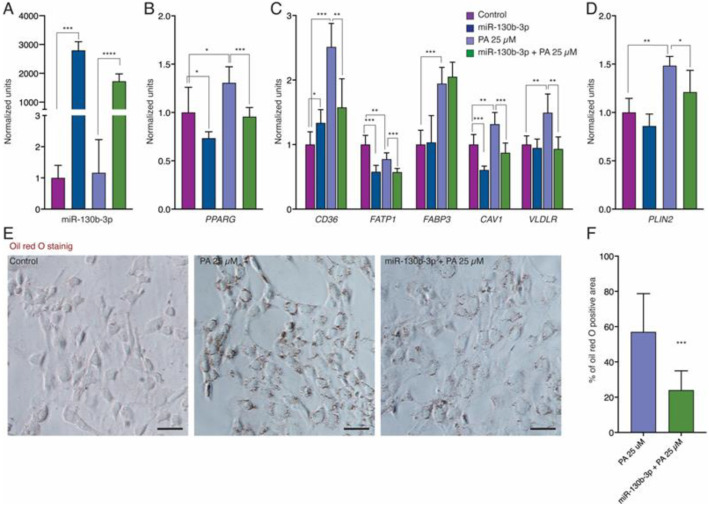
miR-130b-3p regulates FA uptake and storage in cardiomyocytes in controls, PA-treated, and PA-treated plus miR-130b-3p mimic-treated AC16 cells (*n* = 6). (**A**) qRT-PCR analysis of miR-130b-3p expression (**B**) qRT-PCR analysis of *PPARγ* expression. (**C**) qRT-PCR analysis of *CD36*, *FATP1*, *FABP3*, *CAV1* and *VLDLR* expression. (**D**) qRT-PCR analysis of *PLIN2* expression. (**E**) Oil Red O Staining of AC16 exposed to PA and PA plus miR-130b-3p mimic treated and controls. (**F**) Quantification of % of oil red O positive area confirmed the presence of intracellular lipid accumulation in PA-induced AC16 cells. * *p* < 0.05, ** *p* < 0.01, *** *p* < 0.005, **** *p* < 0.0001. Error bars represent SDs. Scale bars = 50 μm. Abbreviations: PA, palmitate.

To investigate the impact of miR-130b-3p on the regulation of *PPARγ* signaling, we created an in vitro lipotoxicity model by culturing AC16 cells in media supplemented with palmitate (PA) for 24 h. To establish the appropriate concentration of PA, we first treated AC16 cells with different PA concentrations, from 25 µM to 100 µM. The MTS assay showed that cell viability dramatically decreased by around 50% with high concentrations of PA, as 50 µM, 75 µM and 100 µM of PA (Figure 1A). Compared to the control group, concentrations above 25 µM of PA altered the cell morphology, changing from an elongated, spindle-like shape to a round shape, significantly reducing the density of live cells (spindle-shaped) and increasing the number of dead cells (round). These effects became more pronounced as the PA dose increased (Figure 1B). Therefore, 25 µM PA was selected as the optimal working concentration for our in vitro model. Once our lipotoxicity model was established, we first found that the expression levels of miR-130b-3p were triggered following overexpression of miR-130b-3p in AC16 cells in both the non-PA group and the PA group (Figure 2A). Then, we examined *PPARγ* expression in AC16 cells in the control condition after miR-130b-3p transfection and after PA treatment. qRT-PCR analysis revealed that miR-130b-3p decreased the expression of *PPARγ*, while PA increased its expression. PA-induced *PPARγ* expression was reverted when miR-130b-3p was overexpressed (Figure 2B).

We therefore analyzed the expression levels of the FA transport marker *CD36*, along with its downstream target genes *FATP1*, *FABP3*, *CAV1*, and *VLDLR*, as well as the fatty acid storage marker *PLIN2*. The expression of *CD36*, *FABP3*, *CAV1*, *VLDRL* and *PLIN2* were upregulated in AC16 cells exposed to PA, whereas *FATP1* was downregulated. miR-130b-3p transfection significantly reverted the PA-induced expression of *CD36*, *CAV1* and *VLDR* but not *FABP3* (Figure 2C). *PLIN2* expression was upregulated with PA and reverted with miR-130b-3p (Figure 2D). Oil Red O staining allows the detection of lipids in cultured cells. We verified the intracellular lipid accumulation in AC16 exposed to PA. However, miR-130b-3p significantly decreases the intracellular lipid content (Figure 2E,F).

### 2.2. miR-130b-3p and Oxidative Stress Triggered by PA

**Figure 3:**
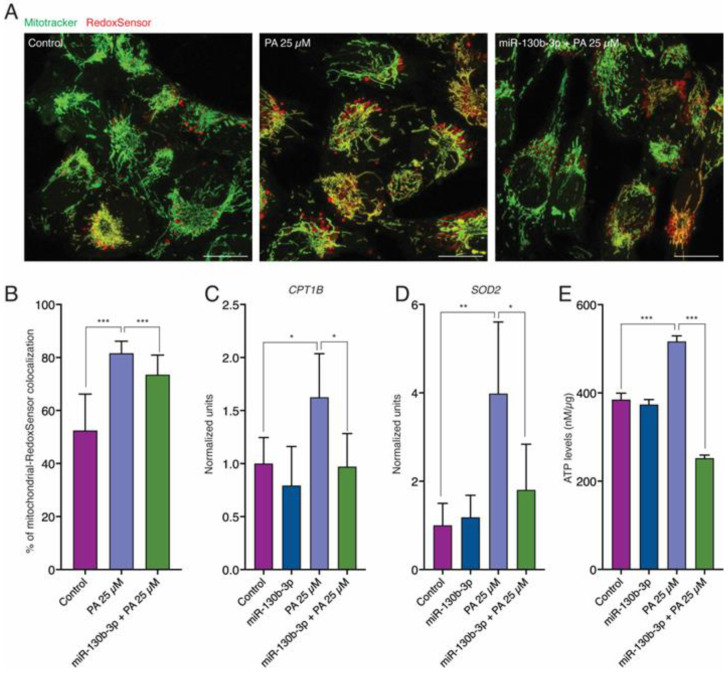
miR-130b-3p alleviates PA-induced mitochondrial oxidation in control, PA-treated and PA-treated plus miR-130b-3p mimic-treated AC16 cells. (**A**) Confocal images showing MitoTracker Green staining and Redox-Sensor co-localization. (**B**) Quantification of MitoTracker Green staining and Redox-Sensor co-localization in AC16 cells (*n* = 24). (**C**) qRT-PCR analysis of *CPT1b* expression in AC16 cells (*n* = 6). (**D**) qRT-PCR analysis of *SOD2* expression in AC16 cells (*n* = 6). (**E**) Total ATP levels in AC16 cells (*n* = 6). * *p* < 0.05, ** *p* < 0.01, *** *p* < 0.005. Error bars represent SDs. Scale bars = 50 μm. Abbreviations: PA, palmitate.

To verify the increase in FAO in PA-treated AC16 cells, we analyzed the oxidative stress intensity based on Mito-Tracker Green and Redox-Sensor Red staining. Confocal images showed greater levels of colocalization in AC16 exposed to PA compared to the control (Figure 3A,B). Consistently, qRT-PCR analysis showed that PA upregulated the expression of *CPT1b*, and mitochondrial superoxide dismutase 2 (*SOD2*). miR-130b-3p overexpression partially rescued the degree of colocalization of Mito-Tracker Green and Redox-Sensor Red (Figure 3A,B). In addition, miR-130b-3p reverted both *CPT1b* and *SOD2* expression levels (Figure 3C,D). We analyzed the total ATP concentration in PA-treated AC16 cells. A significant increase in ATP production was observed in the presence of PA, reverted when miR-130b-3p was overexpressed (Figure 3E).

### 2.3. miR-130b-3p and ER Stress and Apoptosis Induced by PA

**Figure 4:**
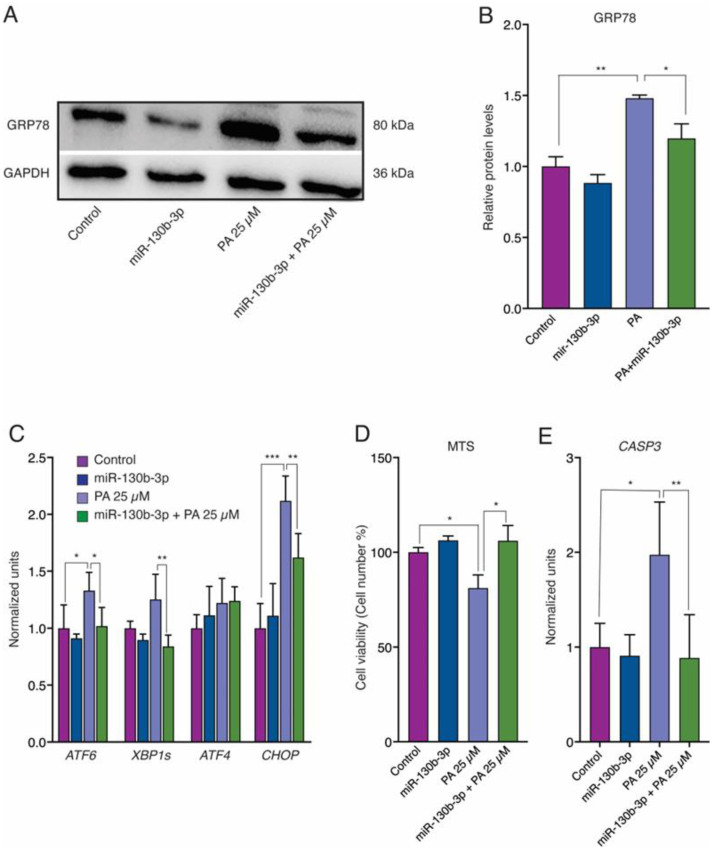
miR-130b-3p reverts PA-induced ER stress and apoptosis in AC16 cells (*n* = 6). (**A**) Representative Western blots of GRP78. (**B**) Densitometry analysis of Western blot. (**C**) qRT-PCR analysis of *ATF6*, *XBP1s*, *ATF4* and *CHOP* expression. (**D**) Cell viability by MTS assays of AC16 cells. (**E**) qRT-PCR analysis of *CASP3* expression in AC16 cells. * *p* < 0.05, ** *p* < 0.01, *** *p* < 0.005. Error bars represent SDs. Abbreviations: PA, palmitate.

We analyzed the expression of ER stress markers in AC16 cells to determine miR-130b-3p influence. Western blot analysis showed a significant increase in GRP78 protein levels after PA treatment (Figure 4A,B). In addition, qRT-PCR analysis showed increased expression of *ATF6*, *XBP1* and *CHOP* but not *ATF4* (Figure 4C). However, when miR-130b-3p was overexpressed, both GRP78 protein levels and *ATF6*, *XBP1* and *CHOP* expression levels were significantly decreased (Figure 4A–C). These results suggest the defensive function of miR-130b-3p against PA-induced ER stress.

We assessed the cell viability and *CASP3* expression and the miR-130b-3p impact on ER stress-induced cardiomyocyte apoptosis. As expected, MTS analysis demonstrated that PA significantly decreased the viability of AC16 cells. In addition, qRT-PCR analysis determined increased expression of *CASP3* (Figure 4D,E). However, miR-130b-3p overexpression reversed cell viability and *CASP3* expression (Figure 4D,E).

### 2.4. miR-130b-3p Directly Targets PPARγ

**Figure 5:**
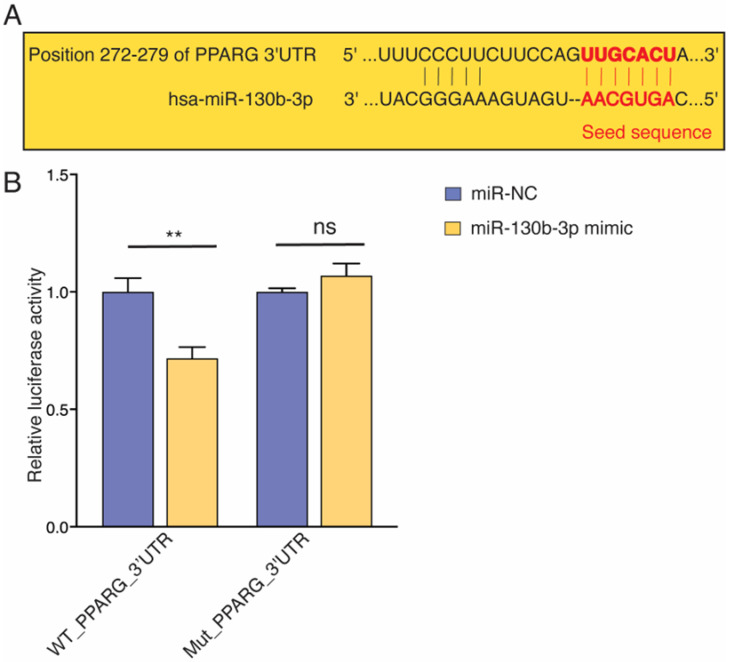
miR-130b-3p targets *PPARγ*. (**A**) Predicted miR-130b-3p binding sites in the 3′UTR of PPARγ. (**B**) Dual-luciferase activity assay in AC16 cells co-transfected with the pMIR-REPORT miRNA expression reporter vector containing the WT or Mut *PPARγ* 3′UTR fragment with miR-130b-3p mimic or the miRNA mimic NC for 24 h. ** *p* < 0.01. Error bars represent SDs. Abbreviations: NC, negative control; WT, wild type; Mut, Mutant; ns, non-significant.

To explore the underlying mechanism by which miR-130b-3p regulates *PPARγ*, we investigated the target of miR-130b-3p. By the prediction of new targets of miR-130b-3p via TargetScan 8.0 (https://www.targetscan.org/vert_80/, accessed on 22 February 2022), we found that the 3´UTR region of human *PPARγ* contained a putative miR-130b-3p target site (Figure 5A). To verify the prediction made through TargetScan that *PPARγ* is a potential target of miR-130b-3p, we performed a dual-luciferase reporter assay. By introducing either wild-type (WT) or the mutant (Mut) binding sequence of *PPARγ* into the luciferase construct and co-transfecting the reporter construct together with miR-130b-3p mimic or miRNA mimic negative control (miR-NC) into T3T cells, we found that miR-130b-3p significantly reduced the luciferase activity of the pMIR-REPORT vector containing WT *PPARγ* 3′UTR but not Mut *PPARγ* 3′UTR (Figure 5B). These results confirmed that *PPARγ* is a direct target of miR-130b-3p.

## 3. Discussion

**Figure 6:**
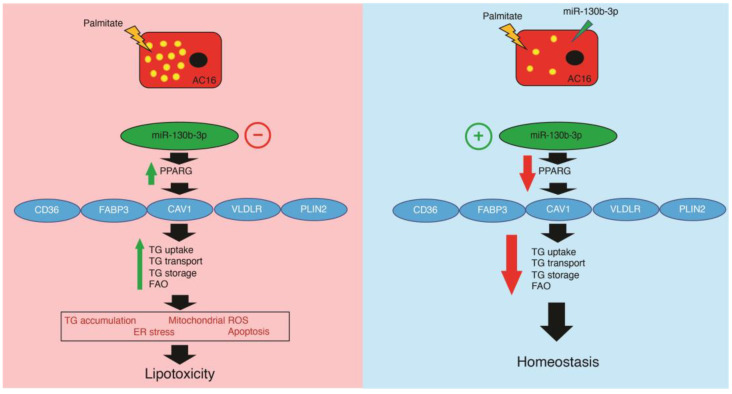
Working model for the role of miR-130b-3p in the regulation of the *PPARγ*-mediated FA uptake in human cardiomyocytes.

We analyze the role of miR-130b-3p in the lipid metabolism of human cardiomyocytes through *PPARγ* expression and its downstream target genes, the mitochondrial oxidative stress status, the ER stress response and the apoptosis induced by PA. We report a critical involvement of miR-130b-3p against lipotoxicity in AC16 cells via regulating *PPARγ* (Figure 6).

Several studies have demonstrated the importance of balanced myocardial lipid metabolism in cardiac function [11,19,20]. *PPARγ* has been extensively studied for its profound impact on gene expression, particularly in the realm of FA metabolism [21]. In this context, mice with *PPARγ* overexpression in cardiomyocytes (*PPARγ*1-transgenic mice) developed DCM, which was associated with increased FA assimilation and lipid storage [22]. Our results demonstrated that exposure to PA in AC16 cells led to the upregulation of *PPARγ*. This was accompanied by increased expression of several genes including *CD36*, *FABP3*, *CAV1*, *VLDLR* and *PLIN2*. Consistent with this, the PA-induced *PPARγ* mRNA expression was previously observed in epithelial-tumorigenic Huh7 cells [23]. By contrast, the downregulation of *FATP1* following by PA treatment, may be a compensatory response to the upregulation of *CD36*. Both FATP1 and CD36 are transmembrane transport proteins, but CD36 is the primary FA receptor in the heart and has a high affinity for long-chain FAs. When CD36 is removed or inhibited, it triggers a compensatory increase in the myocardial expression of *FATP1* [24].

miR-130b-3p has a crucial role in cardiovascular diseases like acute myocardial infarction, myocardial ischemia/reperfusion injury or septic cardiomyopathy [25,26]. Previous studies have confirmed that the *PPARγ* expression is negatively regulated by miR-130b-3p [16,18,27]. This miRNA reduces adipogenesis by repressing *PPARγ* biosynthesis [17,28,29]. Accordingly, we observed that miR-130b-3p overexpression rescued the upregulation of *PPARγ* and its downstream gene targets *CD36*, *CAV1*, *VLDLR* and *PLIN2* in PA-treated AC16 cells. AC16 cells exhibited diminished intracellular lipid accumulation in conditions of miR-130b-3p overexpression, consistent with the reduced FA uptake observed in mice with cardiomyocyte-specific ablation of *CD36* [30]. We also confirmed that *PPARγ* is a direct target of miR-130b-3p in humans. In contrast, the expression of *FABP3* remained unaltered after miR-130b-3p mimic transfection. FABP3 plays an essential role in intracellular FA transport from the cytosolic to the nucleus acting downstream CD36 [31,32]. Moreover, FAPB3 is involved in the uptake of long-chain FAs with CD36. In that process, FAs bind to the inner membrane of FABPc which, in turn, anchors itself by binding to the intracellular part of CD36. For that, upregulate expression of *CD36* which may impact *FABP3* expression [33].

Mitochondrial impairment, a key factor in DCM pathogenesis, drives mitochondrial ROS production [34,35]. PA increases mitochondrial ROS production in different cell types, as muscle cells, endothelial cells, fibroblast or cardiomyocytes. A free FA overload on the mitochondrial electron transport chain potentially increases the generation of electron donors; the excess of electrons causes the conversion into superoxide [36,37]. CPT1b controls the entry of long-chain fatty acyl CoA into mitochondria and SOD2 is an intracellular antioxidant enzyme that defense against ROS-induced oxidative stress and lipid peroxidation. We confirmed increased mitochondrial FAO in AC16 exposed to PA as increased oxidative stress intensity accompanied by higher mitochondrial *CPT1b* and *SOD2* expression. Accordingly, Li et al. that reported that FAO in cardiomyocytes is abrogated by inactivation of *CPT1b* [38]. Also, Garrel et al. demonstrated an increased *SOD2* activity due to FAs’ presence [39]. miR-130b-3p overexpression rescued the PA-induced expression of *CPT1b* and *SOD2*, and lightly alleviated the oxidative stress status in AC16 cells. We did not observe mitochondrial dysfunction in AC16 exposed to PA; total ATP levels were augmented with enhanced mitochondrial FAO. Moreover, ATP levels were reverted by miR-130b-3p overexpression due to the concentration of PA (25 μM) used in our in vitro model compared to the 100–200 μM used in other studies [40,41]. Intrinsic characteristics of each cell’s metabolism may also have an influence. AC16 cell viability has been severely compromised with a higher PA concentration due to cell type-based cytotoxicity [42]. In agreement with this, Kakimoto et al. reported that ATP production is unaltered in a culture cell model of PA overload. They demonstrated that human hepatoma cells exhibit significant flexibility in nutrient utilization, and PA does not impede ATP production [43].

Accumulation of unfolded proteins in the ER lumen leads to ER stress. When ER stress occurs, the three unfolded protein response (UPR) signaling branches named IRE1, PERK, and ATF6 are activated. UPR is also activated under perturbations in cellular FA composition [44]. In this sense, it has been demonstrated that PA induces ER stress-mediated cardiomyocyte death [45]. miR-130b-3p overexpression rescued the PA-increased expression levels of GRP78, a master regulator of UPR. miR-130b-3p presence also decreased mRNA expression of *ATF6* and *XBP1s*, which is downstream of *IRE1*. Our results diverge from previous observations in which PA overload activated *IRE1* and *PERK*, but not *ATF6* in rat cardiomyocytes. This study proposes that the activation of *IRE1* and *PERK* is based on enhanced dimerization consequence of ER membrane impairment, whereas *ATF6* activation does not include the dimerization process [45]. Despite the absence of excessive membrane saturation in our model, we have observed that even a moderate increase in lipid accumulation is sufficient to activate *ATF6*. When the ER stress is severe or prolonged, apoptotic processes are initiated by *CHOP* [46,47]. *ATF6* is an important regulator of *CHOP* [48], a key mediator of ER stress-induced apoptosis [49]. PA upregulated *CHOP* expression and miR-130b-3p reverted it. Accordingly, PA decreased cell viability accompanied by the upregulation of *CASP3*, while this effect was suppressed by miR-130b-3p overexpression.

Our study provides compelling evidence for the critical role of miR-130b-3p in FA metabolism in cardiomyocytes through its interaction with *PPARγ*. Specifically, we have demonstrated that miR-130b-3p modulates FA assimilation and storage in cardiomyocytes by targeting *PPARγ* and that overexpression of miR-130b-3p confers protection to human cardiomyocytes against lipotoxicity. In conclusion, our findings position miR-130b-3p as a promising candidate for therapeutic intervention in lipotoxicity-related DCM. However, additional research is crucial to translate these findings into clinical applications. This study lays the groundwork for future investigations aimed at developing miRNA-based treatments for cardiac lipotoxicity and related cardiomyopathies.

The strengths of our study include novel insights into miR-130b-3p function in cardiac lipid metabolism, a comprehensive examination of cellular processes, and clear mechanistic evidence. However, limitations should be noted. The use of AC16 cells, while consistent with our previous work, may not fully represent primary human cardiomyocytes due to their transformed nature and incomplete maturation. Additionally, the reliance on an in vitro model and the lack of in vivo validation limits the translational potential of our findings. Future research should address these limitations by validating key results in more physiologically relevant models, such as iPSC-derived cardiomyocytes or animal models, to enhance the clinical applicability of our discoveries.

## 4. Materials and Methods

### 4.1. Cell Culture and Transfection

AC16 cells were cultivated in DMEM-F12 supplemented with 10% fetal bovine serum and 1% penicillin/streptomycin and incubated at 37 °C with 5% CO_2_. Cells were treated with 25 µm PA (Cayman Chemical Company, Ann Arbor, MI, USA) for 24 h to create a lipotoxicity in vitro model. The overexpression of miR-130b-3p was performed by transfection of mirVana^®^ has-miR-130-3p mimic (Invitrogen, Carlsbad, CA, USA) with Lipofectamine^®^ RNAiMA Reagent (Invitrogen, Carlsbad, CA, USA), following the manufacturer’s guidelines.

### 4.2. Cellular Oil Red O Staining

AC16 (1 × 110 cells per well) was seeded in a 24-well plate and allowed to attach overnight. Subsequently, the cells were transfected with miR-130b-3p mimic or inhibitor, and/or with PA 25 µM for 24 h, fixed with 4% paraformaldehyde for 15 min, and then stained with Oil red O staining (Bio-Optica, Milan, Italy). After staining, the cells were rinsed with PBS at least three times. Photographs were taken and recorded under a fluorescence inverted microscope, model CKX41, Olympus.

### 4.3. RNA Isolation and Quantitative Real-Time PCR

The total RNA from AC16 cells was isolated using total an RNA Purification Kit (NORGEN, Biotek Corp., Thorold, ON, Canada). mRNA was reverse-transcribed employing a PrimeScript RT reagent Kit (Takara, Shiga, Japan) following the manufacturer’s instructions. qRT-PCR was performed on a CFX96 Real-Time PCR system (Bio-Rad, Hercules, CA, USA) using the iTaq Universal SYBR Green Supermix (Bio-Rad, CA, USA) (primer pairs shown in Supplementary Data). miRNA was reverse-transcribed with miRCURY LNA RT Kit (Qiagen, Hilden, Germany), and miRCURY LNA SYBR Green PCR Kit (Qiagen) for qRT-PCR amplification. mRNA expression was normalized against GAPDH and ß-actin, whereas miRNA expression was normalized against U6 and 5S. The expression levels were calculated by the Livak formula, and the expression of the control group was set to 1.

### 4.4. Western Blotting

Transfected AC16 cells were harvested in RIPA, and protein concentration was determined using a Pierce BCA protein assay kit (Thermo Fisher Scientific, Rockford, IL, USA). Equal amounts of protein (40 µg) were loaded and electrophoresed on 8–12% SDS-PAGE. Membranes were incubated with specific monoclonal anti-GRP78 (BiP) (diluted 1:1000; Cell Signaling Technology, Beverly, MA, USA), and anti-GAPDH antibody (diluted 1:5000; Invitrogen, Rockford, IL, USA) overnight at 4 °C with constant agitation. Following incubation, membranes were washed and incubated for 1 h at room temperature with HRP-linked secondary anti-rabbit IgG (diluted 1:10,000; Cell Signaling, Beverly, MA, USA). After washing, immunoreactive bands were visualized using Clarity Western ECL Substrate (Bio-Rad, Hercules, CA, USA). The immunoreactive bands were analyzed using a lab analysis software imaging densitometer (Bio-Rad, Hercules, CA, USA). The density of each band was evaluated with Image Lab 6.1 analysis software (Bio-Rad, Hercules, CA, USA). β-Actin and GAPDH were used as loading controls, and values were normalized to the signals obtained with control samples.

### 4.5. Oxidative Stress Intensity Assay

Cells were costained with 100 nM MitoTrackerGreen^®^ FM (M7514, Invitrogen, Rockford, IL, USA) and 2.5 µM of RedoxSensor Red CC-1 (R-14060, Invitrogen, Rockford, IL, USA) to determine mitochondrial ROS levels following the manufacture instructions. Briefly, cells were incubated with staining agents at 37 °C for 10 min. After incubation, the cells were rinsed twice with PBS. Cells were imaged using a ZEISS LSM 900 confocal microscope (ZEISS, Oberkochen, Germany). Colocalization was quantified using Image ImageJ 2.9.0 (NIH).

### 4.6. Cell Viability Assay

AC16 cells (5–100 × 10^3^/well) were seeded into a 96-well plate in a final volume of 200 µL/well. Then, we treated them separately with PA and miR-130b-3p mimic to establish the study groups for 24 h in a 5% CO_2_ incubator at 37 °C. The viability of AC16 cells was determined by the ab197010 MTS Cell Proliferation Assay Kit (Abcam, Cambridge, UK). A total of 20 µL per well of MTS reagent was added to each well. Cells were further incubated for three hours at 37 °C in standard culture conditions. The cell viability of each treatment was quantified by determining absorbance in each well at 490 nm using a microplate reader. Images were captured and documented using a light microscope.

### 4.7. Luciferase Reporter Assay

The TargetScan software 8.0 (https://www.targetscan.org/vert_80/, accessed on 22 February 2022) was used to analyze the potential binding sequences within the 3′ untranslated regions (3′ UTR) of *PPARγ* to miR-130b-3p. Then, a mutation was introduced into the potential binding sequences using the Stratagene QuikChange site-directed mutagenesis kit (Agilent Technologies, Santa Clara, CA, USA) but with the enzymes and buffers from the Bio-Rad iPROOF PCR kit (Bio-Rad, CA, USA). The wild-type (WT) or mutant (Mut) binding sequences of PPARG were PCR-amplified and cloned into the pMIR-REPORT vector, and co-transfected with miR-130b-3p mimic and pcLux vector control for internal normalization. Luciferase activity was measured 24 h after transfection by using the Pierce Gaussia Luciferase Flash Assay Kit (Thermo Fisher Scientific, Rockford, IL, USA) and normalized to pcLux vector control by using the Pierce Cypridina Luciferase Flash Assay Kit (Thermo Fisher Scientific, Rockford, IL, USA). In all cases, transfections were carried out in triplicate.

### 4.8. Cellular ATP Level Assay

ATP levels were detected by the ATP Determination Kit (Thermo Fisher Scientific, Rockford, IL, USA). A standard curve was plotted to determine the ATP content of the cells according to the manufacturer’s instructions. Briefly, AC16 cells were treated with PA and miR-130b-3p mimic, for 24 h. The cells lysed were collected in PBS by repeated freezing and thawing. Cell lysates were centrifuged at 12,000× *g* and 4 °C for 5 min to obtain the supernatant. A total of 100 µL of ATP detection solution and 20 µL of supernatant to the 96-well plate were added successively. The chemiluminescence values were determined by a multifunctional fluorescence enzyme marker (Thermo Fisher Scientific, Rockford, IL, USA).

## Data Availability

Data transparency is guaranteed. The datasets generated during and/or analyzed during the current study are available from the corresponding author on rea-sonable request. We used various software for functional enrichment and statistical analysis. All of them are cited in our manuscript.

## Supplementary Materials

The following supporting information can be downloaded at: https://www.mdpi.com/article/10.3390/ijms252212161/s1.
