# Supplementary material for: The Protective Role of miR-130b-3p Against Palmitate-Induced Lipotoxicity in Cardiomyocytes Through PPARγ Pathway
Source: Int J Mol Sci. 2024 Nov 13;25(22):12161. doi: 10.3390/ijms252212161 (PMC11594327; doi:10.3390/ijms252212161)
Supplement: Supplementary file 1 [file ijms-25-12161-s001.zip › ijms-3225269-supplementary.pdf]

SUPPLEMENTARY DATA

Table S1. Quantitative real-time polymerase chain reaction primer pair sequence.

Gene	Forward	Reverse
PPARY	5´-TCCAACTCCCTCATGGCAATTG-3´	5´-ATGAGACATCCCCACTGCAAG-3´
CD36	5´-CTCTTTCCTGCAGCCCAATG-3´	5´-CTGCCACAGCCAGATTGAGA-3´
PLIN2	5´-GATGAGTCCCACTGTGCTGA-3´	5´-GCATTGCGGAACACTGAGTA-3´
GAPDH	5´-AGCCACATCGCTCAGACAC-3´	5´-AATACGACCAAATCCGTTGACT-3´
B-actin	5´-TGTGGCATCCACGAAACTACC-3´	5´-CTCAGGAGGAGCAATGATCTTGAT-3´
CPT1B	5´-GACTGTGCGTTCCTGTACCA-3´	5´-CTTCTTGATGAGGCCTTTGC-3´
FATP1	5´-TACCAGGAGCTGCAGAAGGT-3´	5´-CGAGCAGATGCGAGTGTAGA-3´
CAV1	5´-GAGCTGAGCGAGAAGCAAGT-3´	5´-CAGCAAGCGGTAAAACCAGT-3´
FABP3	5´-AGCATGACCAAGCCTACCAC-3´	5´-GGTGAACAAGTTTCCCTCCA-3´
VLDLR	5´-ATGGGCCATTCTTCCTCTCT-3´	5´-ACGTGTGTCCAACAGAAGCA-3´
CHOP	5´ -TCACCACACCTGAAAGCAGA-3´	5´-TCTTGCAGGTCCTCATACCA-3´
ATF6	5´ -AATACTGAACTATGGACCTATGAGCA-3´	5´ -TTGCAGGGCTCACACTAGG-3´
XBP1	5´-TGCGTAGTCTGGAGCTATGGT-3´	5´-CCCGACAGAAGCAGAACTTT-3´
SOD2	5´-GGCTGTAGCACCAGGTCAG-3´	5´- TGTGTAGGAGAGGGGCGTAT-3´
CASP3	5´-TGAGTGCTCGCAGCTCATA-3´	5´-GGGCTCGCTAACTCCTCAC-3´
ATF4	5´-TCTCCAGCGACAAGGCTAA-3´	5´-CCAATCTGTCCCGGAGAA-3´

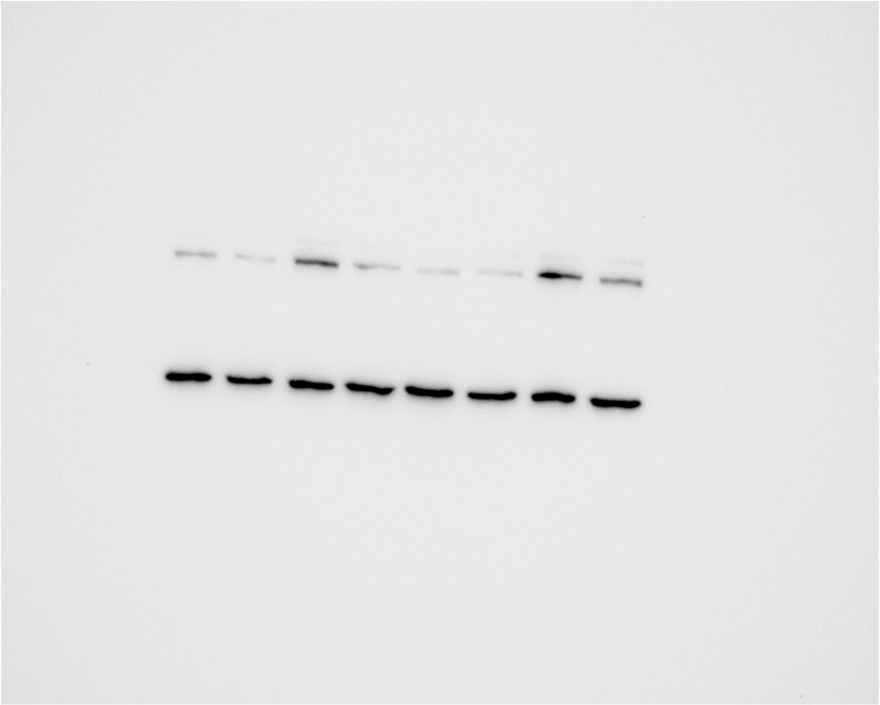

Figure S1. Uncropped and unmodified Western blot of GAPDH

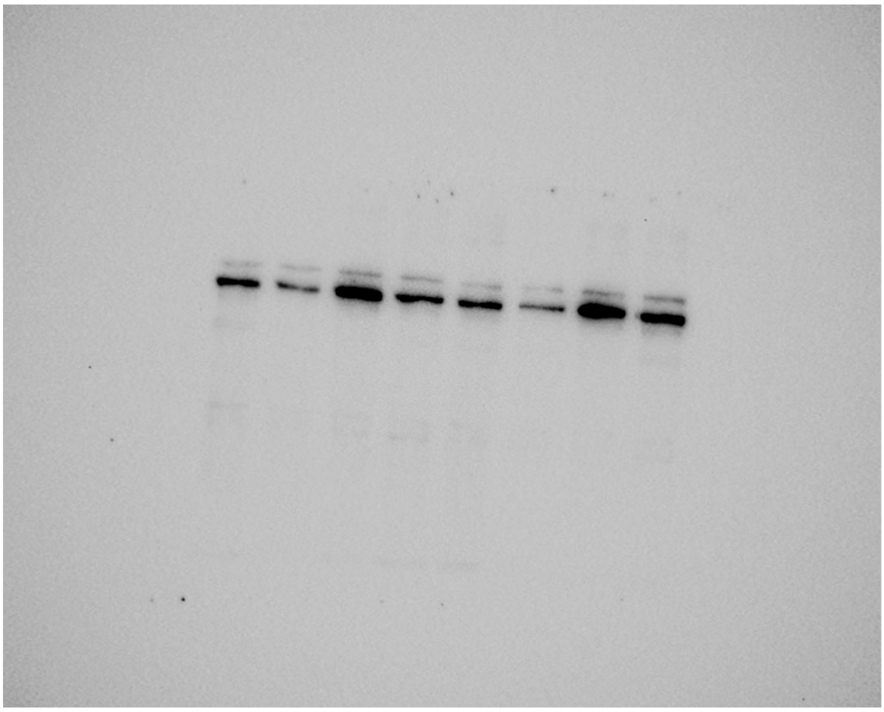

Figure S2. Uncropped and unmodified Western blot of GRP78
